# Dose-Dependent Efficacy and Safety of Tirzepatide for Weight Loss in Non-diabetic Adults With Obesity: A Systematic Review and Meta-analysis of Randomized Controlled Trials

**DOI:** 10.7759/cureus.85531

**Published:** 2025-06-07

**Authors:** Alousious Kasagga, Amanuel Kefyalew Assefa, Maysaa N Amin, Rahma Hashish, Khaled Agha Tabari, Shivling S Swami, Kevin Nakasagga

**Affiliations:** 1 Pathology, Peking University, Beijing, CHN; 2 Orthopaedics and Trauma, University Hospitals of Leicester NHS Trust, Leicester, GBR; 3 Microbiology/Immunology, California Institute of Behavioral Neurosciences and Psychology, Fairfield, USA; 4 General Medicine, Sherwood Forest Hospitals NHS Foundation Trust, Nottingham, GBR; 5 Radiology, Queen Elizabeth University Hospital, Glasgow, GBR; 6 Internal Medicine, California Institute of Behavioral Neurosciences and Psychology, Fairfield, USA; 7 Anaesthesiology, Milwaukee School of Engineering, Milwaukee, USA

**Keywords:** dose response, dual incretin therapy, gip/glp-1 agonist, meta-analysis, meta-regression, non-diabetic adults, obesity treatment, systematic review, tirzepatide, weight loss therapy

## Abstract

Tirzepatide, a dual glucose-dependent insulinotropic polypeptide (GIP)/glucagon-like peptide-1 (GLP-1) receptor agonist, has shown promising weight-loss effects in previous trials. However, its dose-dependent efficacy and safety in obese adults without diabetes have not been fully defined. We conducted a systematic review and meta-analysis of randomized controlled trials (RCTs) evaluating tirzepatide in obese adults without diabetes. PubMed, Cochrane CENTRAL, and ClinicalTrials.gov were searched from January 2020 to April 2025. Eligible studies compared tirzepatide (5-15 mg or maximum tolerated dose (MTD)) to a placebo over a minimum duration of 52 weeks.

The primary outcome was the percent change in body weight. Secondary outcomes included changes in body mass index (BMI), waist circumference, hemoglobin A1c (HbA1c), systolic blood pressure (SBP), quality-of-life scores (Short-Form Health Survey Version 2 (SF-36v2) and the Impact of Weight on Quality of Life-Lite Clinical Trials version (IWQOL-Lite-CT)), and achievement of categorical weight-loss thresholds. Safety outcomes included the incidence of adverse events. Data were pooled using fixed- or random-effects models, and dose-response meta-regression was performed.

Four RCTs (n = 3,553 participants) met the inclusion criteria. Tirzepatide significantly reduced the percentage body weight compared to placebo (mean difference -16.54%; 95% CI -17.48 to -15.59; p < 0.00001), with a clear dose-response relationship (meta-regression β = -0.72% per 1 mg increase; p = 0.0014). Participants receiving tirzepatide were markedly more likely to achieve clinically meaningful weight-loss thresholds, such as ≥15% (OR 23.25; 95% CI 18.06-29.94). Tirzepatide also improved BMI (-7.09 kg/m²), waist circumference (-12.77 cm), HbA1c (-0.42%), and quality-of-life scores. Gastrointestinal adverse events, including nausea (OR 4.20), vomiting (OR 6.93), and diarrhea (OR 3.80) were more frequent with tirzepatide; however, the rate of serious adverse events was comparable to placebo (OR 0.97). Sensitivity analyses confirmed the reliability of the findings.

In conclusion, tirzepatide produces substantial, dose-dependent weight loss and improves metabolic and quality-of-life outcomes in obese adults without diabetes. Despite a higher incidence of gastrointestinal side effects, the lack of increased serious adverse events supports its favorable risk-benefit profile for long-term obesity treatment.

## Introduction and background

Obesity is a chronic, relapsing condition that affects an estimated 890 million adults worldwide and contributes significantly to global illness, death, and healthcare costs [1]. While lifestyle changes remain the foundation of obesity treatment, achieving and maintaining long-term weight loss are often challenging in real-world settings, with high rates of weight regain and poor adherence [2]. Bariatric surgery can lead to substantial and lasting weight loss, but its cost, invasiveness, and strict eligibility requirements limit its accessibility for most people with obesity [3,4]. As a result, there is growing interest in pharmacologic treatments that can deliver meaningful weight loss with a favorable safety and tolerability profile.

Among available treatments, glucagon-like peptide-1 (GLP-1) receptor agonists have become a cornerstone of obesity pharmacotherapy. Medications like liraglutide and semaglutide, originally developed for the treatment of type 2 diabetes, have demonstrated effectiveness in promoting weight loss and improving cardiometabolic risk factors, leading to their expanded use in the management of obesity. However, in non-diabetic individuals, weight loss outcomes tend to be more modest, and gastrointestinal side effects often limit long-term use [5]. These challenges have prompted the development of dual-incretin therapies that target both the GLP-1 and glucose-dependent insulinotropic polypeptide (GIP) receptors to enhance metabolic regulation and appetite suppression [6].

Tirzepatide, a novel dual GIP/GLP-1 receptor agonist, was first approved for improving glycemic control in type 2 diabetes and has shown superior weight-loss effects compared to GLP-1 monotherapy [6,7]. Recent phase 3 SURMOUNT trials have extended this evidence to obese adults without diabetes, suggesting that tirzepatide may offer promising, even transformative, outcomes in this population [7-10]. Despite this, no meta-analysis has yet synthesized data from randomized controlled trials (RCTs) specifically evaluating tirzepatide’s efficacy and safety in non-diabetic adults with obesity, nor explored its dose-dependent effects on weight loss and quality-of-life measures.

In this study, we conducted a systematic review and meta-analysis of RCTs that assess tirzepatide in non-diabetic adults with obesity. Our goals were to quantify dose-dependent weight loss, evaluate changes in cardiometabolic and quality-of-life outcomes, and assess safety and tolerability, including adverse events and treatment discontinuation. The results provide clinically meaningful pooled estimates to inform evidence-based decisions in the treatment of non-diabetic obesity.

## Review

Methods

Search Strategy

We conducted a comprehensive literature search using PubMed, Cochrane CENTRAL, and ClinicalTrials.gov for studies published between January 1, 2020, and April 25, 2025. The search strategy included terms related to tirzepatide and obesity-related weight loss. For tirzepatide, we used the keywords “tirzepatide” and “LY3298176”. For the obesity and weight loss concept, we included keywords such as “obesity”, “overweight”, and “weight loss”, along with corresponding MeSH terms: “Obesity” (MeSH) and “Weight Loss” (MeSH). To identify RCTs, we used the terms “randomized controlled trial” and “RCT” and applied database-specific filters for study type where available.

Search strings were constructed using Boolean operators (AND, OR) to combine these concepts. Searches were limited to human studies, English-language publications, and RCTs. Because diabetic status is often reported within study eligibility criteria rather than in titles or abstracts, we did not include terms related to “non-diabetic” populations in the search. Instead, studies enrolling participants with diabetes were excluded during the screening process. We did not include gray literature or conference abstracts. Additionally, the reference lists of included studies were manually reviewed to identify any further eligible publications. This systematic review and meta-analysis were conducted following the PRISMA 2020 guidelines [11].

Eligibility Criteria

Studies were included if they met the following criteria: (1) RCT design; (2) enrolled adults aged ≥18 years with obesity, as defined by each study (e.g., body mass index (BMI) ≥ 30 kg/m² or clinical diagnosis); (3) investigated tirzepatide at any dosage compared to placebo or another tirzepatide dosage; and (4) had a minimum treatment duration of 52 weeks. Studies were excluded if they enrolled individuals with diagnosed type 2 diabetes mellitus. However, studies including prediabetic participants without a formal diabetes diagnosis were eligible. The primary outcome was a change in body weight. Secondary outcomes included changes in waist circumference, BMI, hemoglobin A1c (HbA1c), systolic blood pressure (SBP), and quality-of-life scores (Short-Form Health Survey version 2 (SF-36v2) and the Impact of Weight on Quality of Life-Lite Clinical Trials version (IWQOL-Lite-CT)). We also assessed the proportions of participants achieving ≥5%, ≥10%, ≥15%, ≥20%, and ≥25% weight loss. Safety outcomes included the occurrence of any adverse events, serious adverse events, treatment discontinuations because of adverse events, and specific adverse events such as nausea, vomiting, diarrhea, and nasopharyngitis. We excluded non-randomized trials, observational studies, review articles, conference abstracts, non-human studies, and any studies that included participants with type 2 diabetes.

Study Selection

Three reviewers separately evaluated titles, abstracts, and full-text articles using a manual procedure in Microsoft Excel (Microsoft Corp., Redmond, WA, US). The evaluation occurred in two stages: a preliminary review of titles and abstracts, followed by an in-depth review of full texts according to the established eligibility criteria. Any differences in judgment were addressed through discussion and agreement.

Data Extraction

Two reviewers independently gathered information utilizing a standardized extraction form. Extracted variables included study characteristics (first author, year, country, trial name), participant demographics (sample size, mean age, sex distribution, baseline BMI), intervention details (tirzepatide dosage and treatment duration), efficacy outcomes (change in body weight, BMI, waist circumference, HbA1c, SBP, IWQOL-Lite, and SF-36v2 scores and proportions achieving ≥5%, ≥10%, ≥15%, ≥20%, and ≥25% weight loss), and safety outcomes (any adverse events, serious adverse events, discontinuations due to adverse events, and specific adverse events including nausea, vomiting, diarrhea, and nasopharyngitis). For studies reporting multiple-tirzepatide-dose arms, data were extracted separately for each dose group.

Risk of Bias Assessment

Two independent reviewers evaluated the risk of bias for every study included using the Cochrane Risk of Bias 2.0 tool [12]. Assessments were carried out at the level of the study across five areas: randomization process, deviations from intended interventions, missing outcome data, measurement of outcomes, and selection of reported results. Each area was classified as “low risk,” “some concerns,” or “high risk.” The risk of bias evaluations were represented visually using robvis [13].

Statistical Analysis

All meta-analyses were conducted using RevMan version 5.4.1 (The Cochrane Collaboration, London, England, UK). Forest plots were generated using a fixed-effects model based on the assumption of low between-study heterogeneity due to the consistency of interventions and populations. Continuous outcomes were combined using mean differences (MDs) along with 95% CIs, whereas dichotomous outcomes were aggregated using risk ratios (RRs) accompanied by 95% CIs. The I² statistic and Cochran’s Q test were employed to evaluate heterogeneity, which was classified as low (<25%), moderate (25%-50%), or substantial (>50%). Sensitivity analyses were performed using a leave-one-out approach to assess the robustness of pooled estimates.

A dose-response meta-regression was conducted using a random-effects model in R version 4.5.0 (R Foundation for Statistical Computing, Vienna, Austria, https://www.R-project.org/) via RStudio (Posit Software, PBC, Boston, MA, US) with the metafor package [14]. A linear meta-regression model was used to evaluate the relationship between tirzepatide dosage (in milligrams) and change in body weight. Subgroup analyses were performed for tirzepatide dosage groups of 5 mg, 10 mg, 15 mg, and maximum tolerated dose (MTD; defined as 10-15 mg). The existence of publication bias was evaluated through a visual examination of funnel plots. Given the small number of studies included, formal statistical analyses like Egger’s regression were not performed.

Results

Study Selection

**Figure 1:**
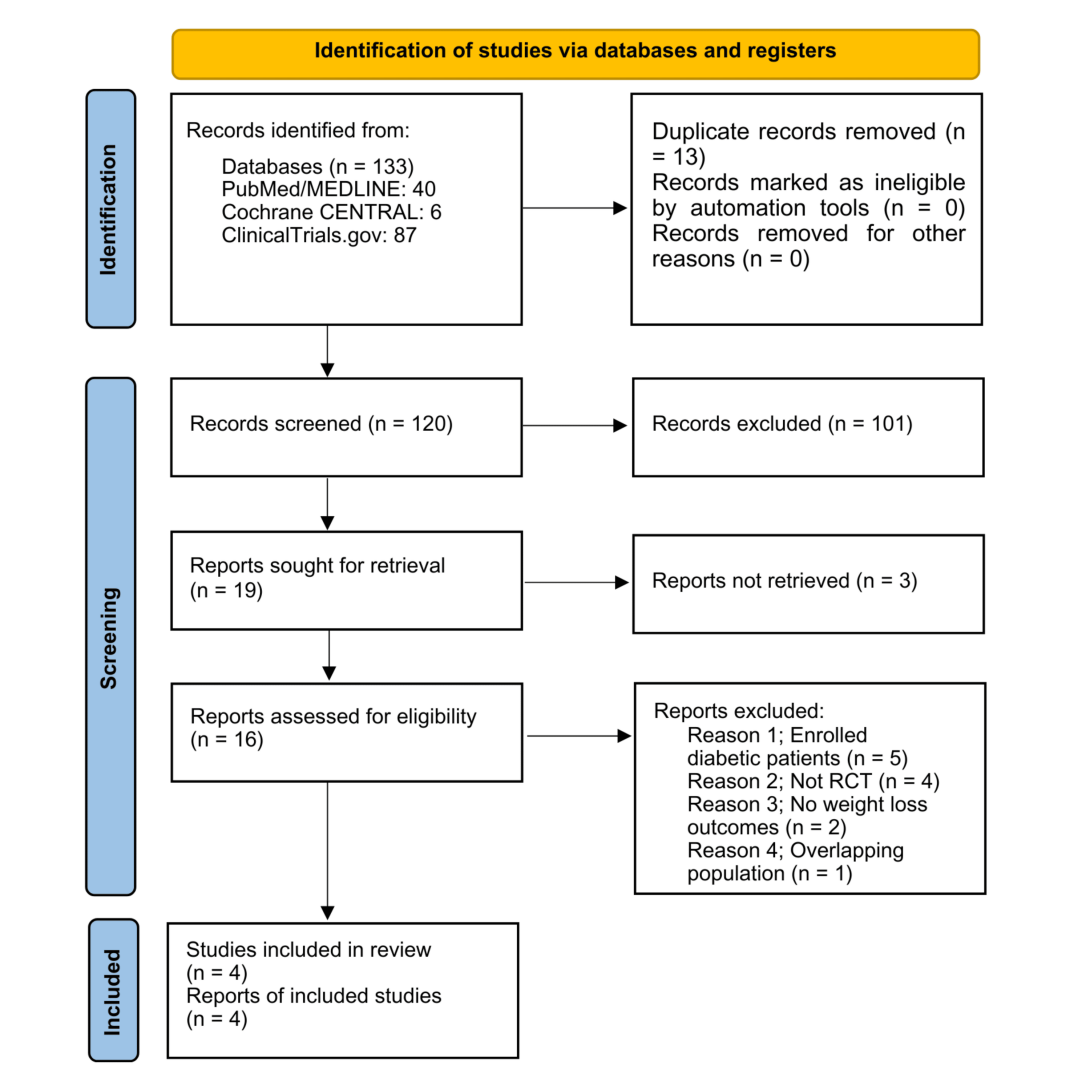
PRISMA flowchart of the study selection process PRISMA: Preferred Reporting Items for Systematic Reviews and Meta-Analyses.

A total of 133 studies were identified through database searches (PubMed, 40; Cochrane CENTRAL, 6; ClinicalTrials.gov, 87). After removing 13 duplicates, 120 unique studies remained for the title and abstract screening. Of these, 101 were excluded for not meeting the inclusion criteria. Nineteen full-text articles were retrieved and assessed for eligibility. Twelve were excluded for the following reasons: inclusion of participants with type 2 diabetes (n = 5), non-randomized study design (n = 4), lack of weight loss outcomes (n = 2), and overlapping study populations (n = 1). In the end, four RCTs [7-10] met the eligibility criteria and were included in both the qualitative and quantitative syntheses (Figure 1).

Study Characteristics

All studies involved were randomized, double-blind, placebo-controlled trials assessing the effectiveness and safety of tirzepatide in obese adults without diabetes. Sample sizes for individual treatment arms ranged from 70 to 636 participants. The mean baseline age of participants ranged from 34.7 to 52.3 years, and the mean baseline BMI ranged from 32.0 to 38.2 kg/m². Tirzepatide was administered subcutaneously in all studies at varying dosages, including 5 mg, 10 mg, 15 mg, and MTD (10-15 mg). Several trials included multiple-tirzepatide-dose groups. In all studies, the comparator was a placebo. The primary endpoint in each trial was a change in body weight from baseline. Study durations ranged from 52 to 72 weeks. All trials were sponsored by Eli Lilly and Company and registered on ClinicalTrials.gov (see Table 1).

**Table 1:** Characteristics of included studies RCT: randomized controlled trial, T: treatment group, C: comparator group, M/F: male/female, MTD: maximum tolerated dose. SURMOUNT, SURMOUNT-CN, and SURMOUNT-J refer to individual phase 3 randomized controlled trials evaluating tirzepatide for chronic weight management in global, Chinese, and Japanese adult populations, respectively.

Author (year)	Study design	Trial name	Treatment	Sample size (T/C)	Comparator	Mean age (T/C)	Sex (M/F) (T/C)	Baseline weight (T/C)	Baseline BMI (T/C)	Primary outcome	Follow-up	Blinding	Funding	Trial registration
Jastreboff et al. (2022) [7]	RCT	SURMOUNT-1	Tirzepatide 5 mg	630/643	Placebo	45.6 (12.7); 44.4 (12.5)	206/426; 207/436	102.9 (20.7); 104.8 (21.4)	37.4 (6.6); 38.2 (6.9)	Weight loss	72 weeks	Double-blind	Eli Lilly and Company	NCT04184622
			Tirzepatide 10 mg	636/643		44.7 (12.4); 44.4 (12.5)	209/427; 207/436	105.8 (23.3); 104.8 (21.4)	38.2 (7.0); 38.2 (6.9)					
			Tirzepatide 15 mg	630/643		44.9 (12.3); 44.4 (12.5)	205/425; 207/436	105.6 (22.9); 104.8 (21.4)	38.1 (6.7); 38.2 (6.9)					
Wadden et al. (2023) [8]	RCT	SURMOUNT-3	Tirzepatide 10-15 mg (MTD)	287/292	Placebo	45.4 (12.6); 45.7 (11.8)	106/181; 109/183	102.5 (22.1); 101.3 (20.7)	36.1 (6.1); 35.7 (6.4)	Weight loss	72 weeks	Double-blind	Eli Lilly and Company	NCT04657016
Kadowaki et al. (2025) [9]	RCT	SURMOUNT-J	Tirzepatide 10 mg	73/75	Placebo	49.0 (10.9); 52.3 (10.9)	43/30; 45/30	92.4 (15.0); 92.0 (15.3)	33.2 (4.1); 33.7 (4.9)	Weight loss	72 weeks	Double-blind	Eli Lilly and Company	NCT04844918
			Tirzepatide 15 mg	77/75		51.1 (10.3); 52.3 (10.9)	45/32; 45/30	91.7 (14.8); 92.0 (15.3)	33.6 (4.3); 33.7 (4.9)					
Zhao et al. (2024) [10]	RCT	SURMOUNT-CN	Tirzepatide 10 mg	70/69	Placebo	34.7 (7.2); 37.8 (10.2)	35/35; 36/33	92.2 (16.2); 92.0 (15.8)	32.6 (4.1); 32.4 (3.6)	Weight loss	52 weeks	Double-blind	Eli Lilly and Company	NCT05024032
			Tirzepatide 15 mg	71/69		35.8 (9.3); 37.8 (10.2)	36/35; 36/33	91.3 (16.2); 92.0 (15.8)	32.0 (3.7); 32.4 (3.6)					

Risk of Bias Assessment

**Figure 2:**
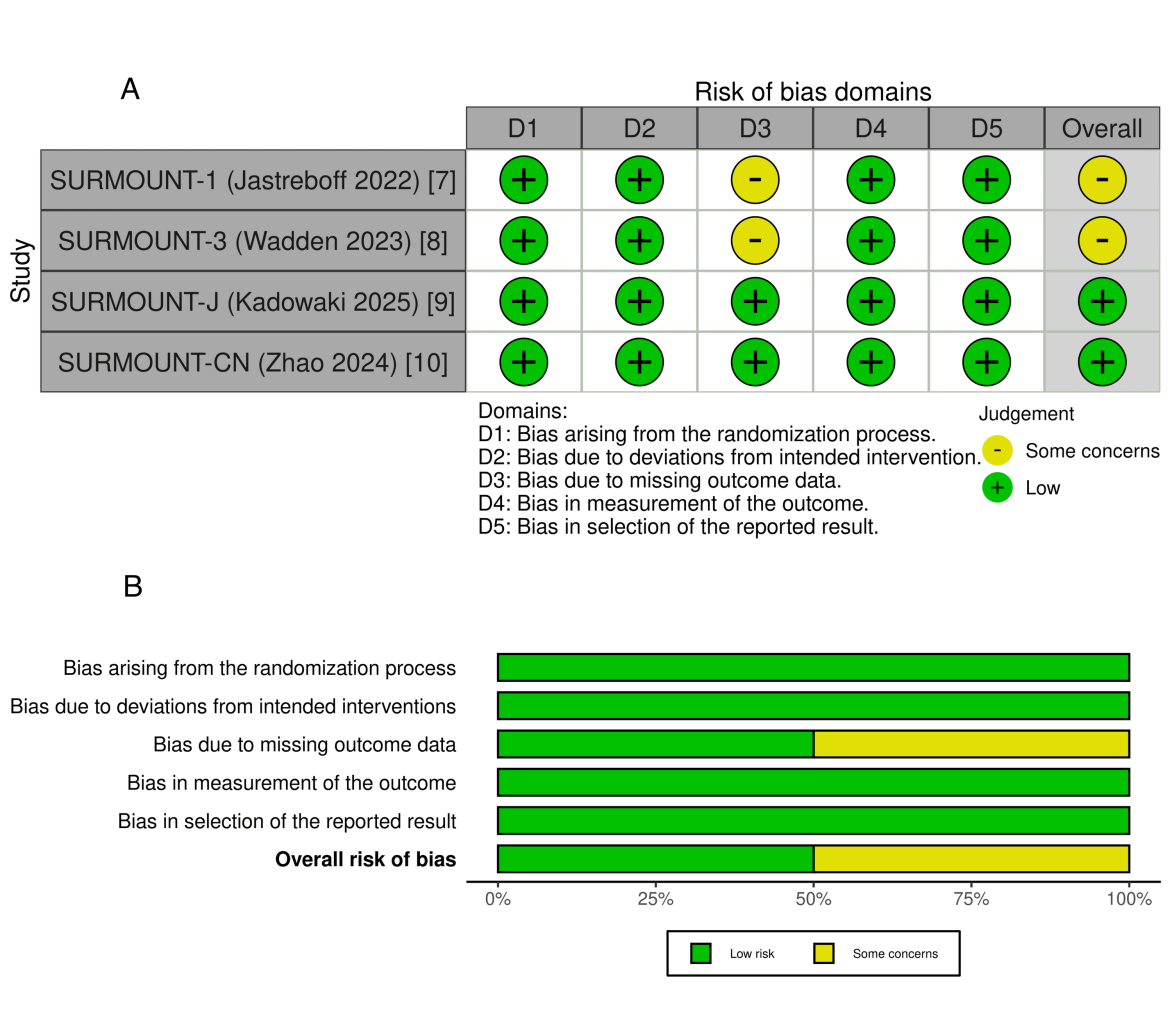
(A) Risk of bias assessment across studies. (B) Summary of risk of bias proportions across domains SURMOUNT, SURMOUNT-CN, and SURMOUNT-J refer to individual phase 3 randomized controlled trials evaluating tirzepatide for chronic weight management in global, Chinese, and Japanese adult populations, respectively. Note: This figure was generated using robvis, a visualization tool based on the Cochrane Risk of Bias 2.0 tool, both available under the Creative Commons Attribution 4.0 License (CC BY 4.0).

The risk of bias was evaluated using the Cochrane Risk of Bias 2.0 tool. Both SURMOUNT-J [9] and SURMOUNT-CN [10] were rated as having a low risk of bias across all domains. SURMOUNT-1 [7] and SURMOUNT-3 [8] received an overall rating of some concerns, primarily due to missing outcome data (D3), reflecting higher attrition rates in placebo groups compared to those receiving tirzepatide. All other domains, including randomization, blinding, outcome measurement, and selective reporting, were rated as low risk across all included studies. These findings are shown in Figure 2.

Percent Weight Change

The primary efficacy outcome was the percent change in body weight from baseline. Tirzepatide treatment demonstrated significantly greater weight reduction compared to placebo across all evaluated doses, with a pooled MD of -16.54% (95% CI -17.48% to -15.59%; p < 0.00001). Substantial heterogeneity was observed across studies (I² = 85%, p < 0.00001).

**Figure 3:**
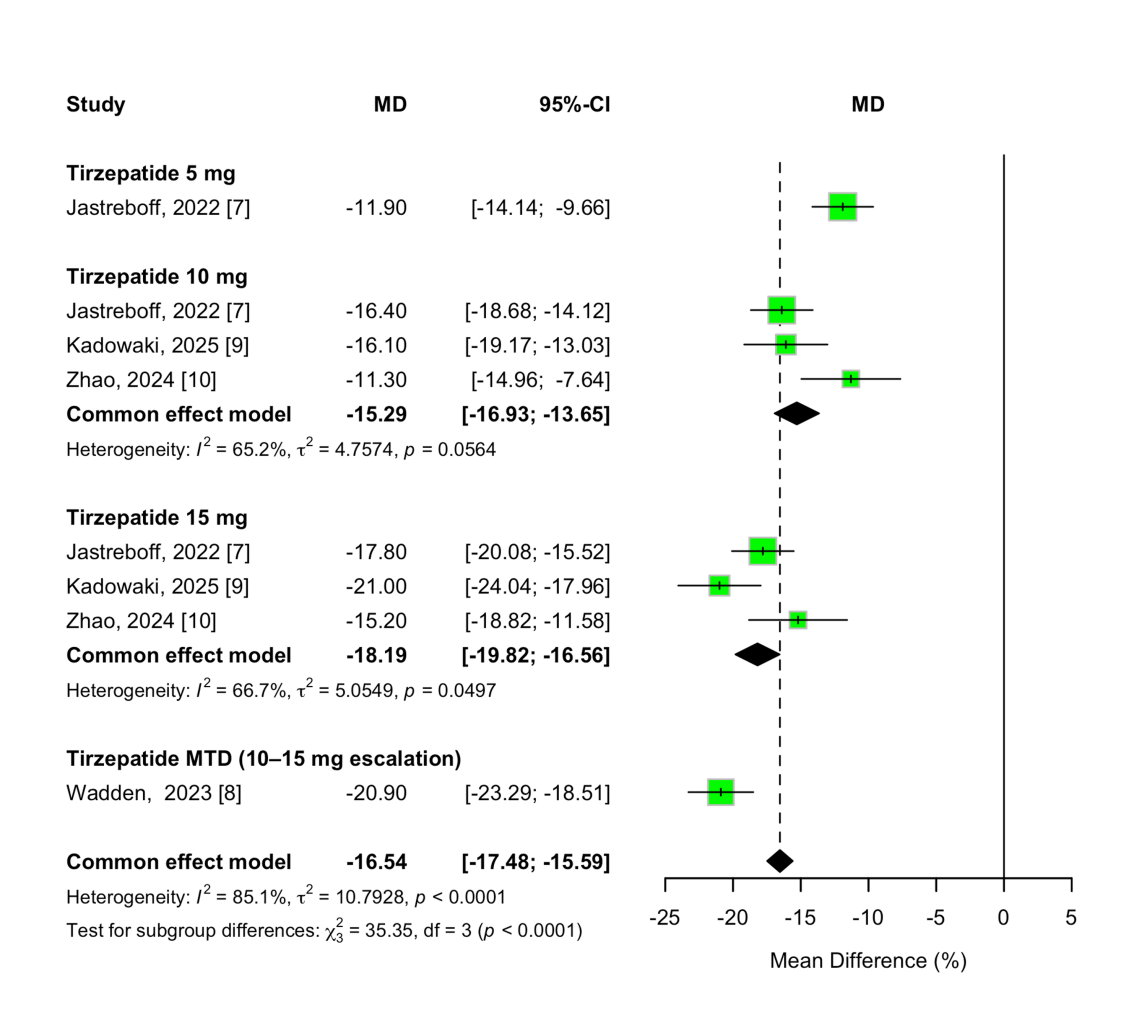
Forest plot for percent weight change

A dose-dependent pattern was evident, with higher tirzepatide doses producing greater weight loss. Treatment with 5 mg resulted in a mean reduction of -11.90% (95% CI -14.14% to -9.66%; p < 0.00001), while the 10 mg and 15 mg dose treatments led to reductions of -15.29% (95% CI -16.93% to -13.65%) and -18.19% (95% CI -19.82% to -16.56%), respectively. Participants who escalated to their MTD (10 or 15 mg) experienced the greatest reduction, with a pooled MD of -20.90% (95% CI -23.29% to -18.51%; p < 0.00001). A formal test for subgroup differences confirmed statistically significant variation across doses (χ² = 35.35, df = 3, p < 0.00001; I² = 91.5%). These results are demonstrated in Figure 3.

**Figure 4:**
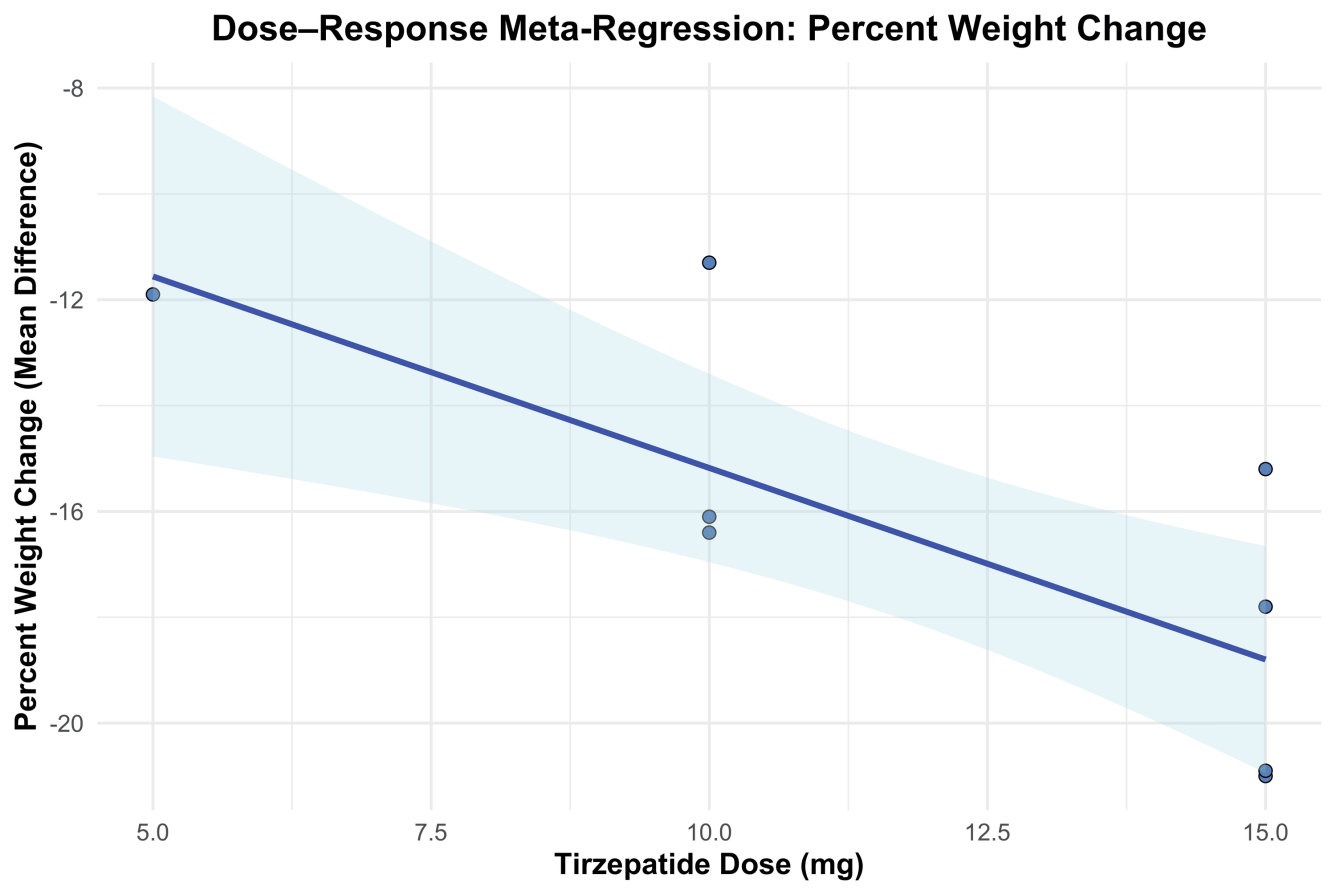
Meta-regression plot for percent weight change

To further investigate the dose-response relationship, a meta-regression analysis was performed. Results indicated that each 1 mg increase in tirzepatide dosage was associated with an additional 0.72% reduction in body weight from baseline (β = -0.7239; 95% CI -1.1673 to -0.2804; p = 0.0014). The model accounted for 69.66% of the between-study heterogeneity (R²), with a significant moderator effect (QM(1) = 10.23, p = 0.0014). Despite this, residual heterogeneity remained statistically significant (QE(6) = 15.15, p = 0.0191), suggesting that some variability was not explained by dose alone. The dose-response trend is illustrated in Figure 4.

BMI Change

Change in BMI from baseline was evaluated as a secondary efficacy outcome. Tirzepatide treatment led to significant reductions in BMI compared to placebo across all studied doses. The overall MD was -7.09 kg/m² (95% CI -7.48 to -6.70; p < 0.00001), favoring tirzepatide. Substantial heterogeneity was observed among studies (I² = 96%, p < 0.00001).

**Figure 5:**
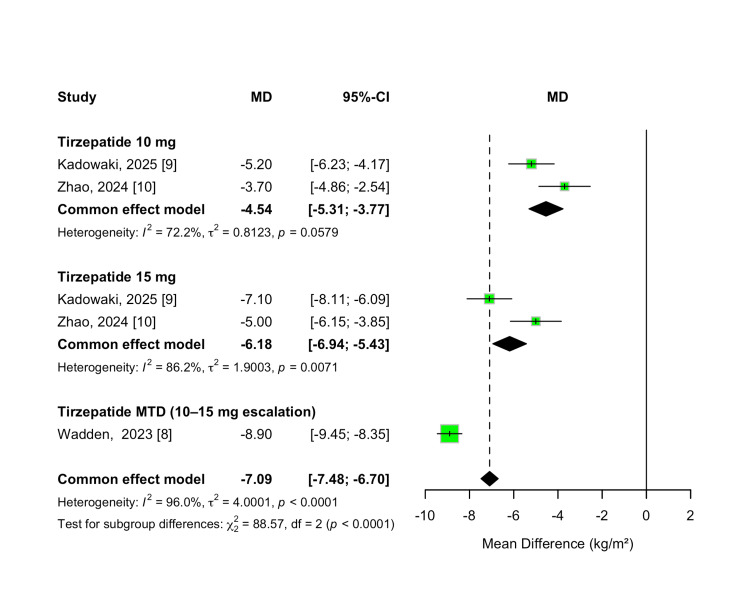
Forest plot for BMI change

A dose-dependent pattern was again evident, with greater BMI reductions observed at higher tirzepatide doses. Treatment with a 10 mg dose resulted in a mean reduction of -4.54 kg/m² (95% CI -5.31 to -3.77; p < 0.00001), while the 15 mg dose yielded a reduction of -6.18 kg/m² (95% CI -6.94 to -5.43). Participants who escalated to their MTD (10 or 15 mg) experienced the greatest reduction, with a pooled MD of -8.90 kg/m² (95% CI -9.45 to -8.35; p < 0.00001). A formal test for subgroup differences confirmed statistically significant variation in BMI reduction across dose levels (χ² = 88.57, df = 2, p < 0.00001; I² = 97.7%). These results are shown in Figure 5.

**Figure 6:**
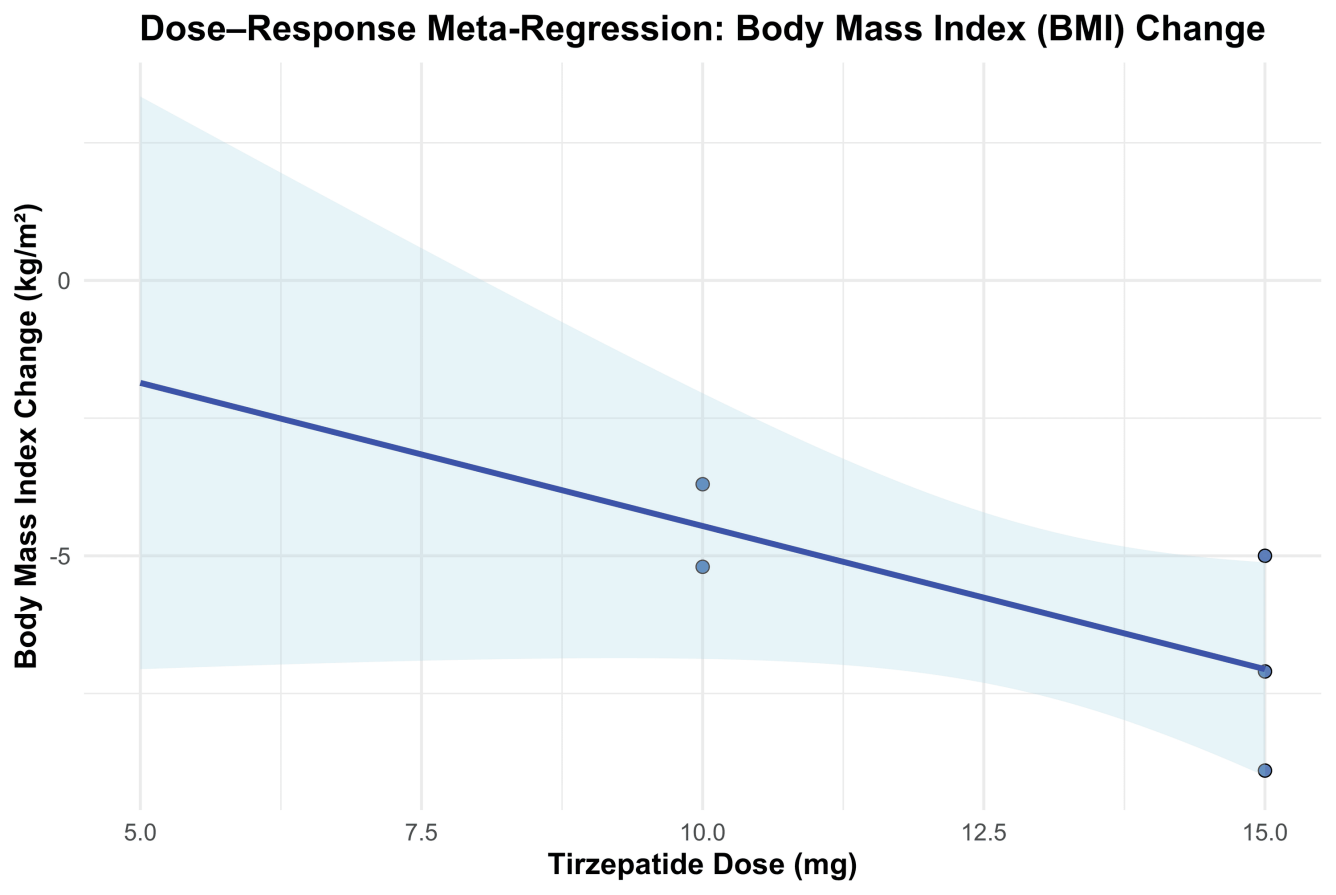
Meta-regression plot for BMI change

To further explore the dose-response relationship for BMI reduction, a meta-regression analysis was performed. Each 1 mg increase in tirzepatide dosage was associated with an additional 0.52 kg/m² reduction in BMI; however, this association did not reach statistical significance (β = -0.5199; 95% CI -1.1390 to 0.0992; p = 0.0998). The model accounted for 32.14% of the between-study heterogeneity (R²), but the dose effect remained non-significant (QM(1) = 2.71, p = 0.0998). Residual heterogeneity was still statistically significant (QE(3) = 43.05, p < 0.0001), indicating that unexplained variability persisted. The dose-response relationship is presented in Figure 6.

Waist Circumference Change

Waist circumference change from baseline was evaluated as a secondary efficacy outcome to assess the reduction in central adiposity. Tirzepatide treatment resulted in significantly greater waist circumference reductions than placebo across all evaluated doses. The overall MD was -12.77 cm (95% CI -13.66 to -11.89; p < 0.00001), favoring tirzepatide, with substantial heterogeneity across studies (I² = 70%, p = 0.002).

**Figure 7:**
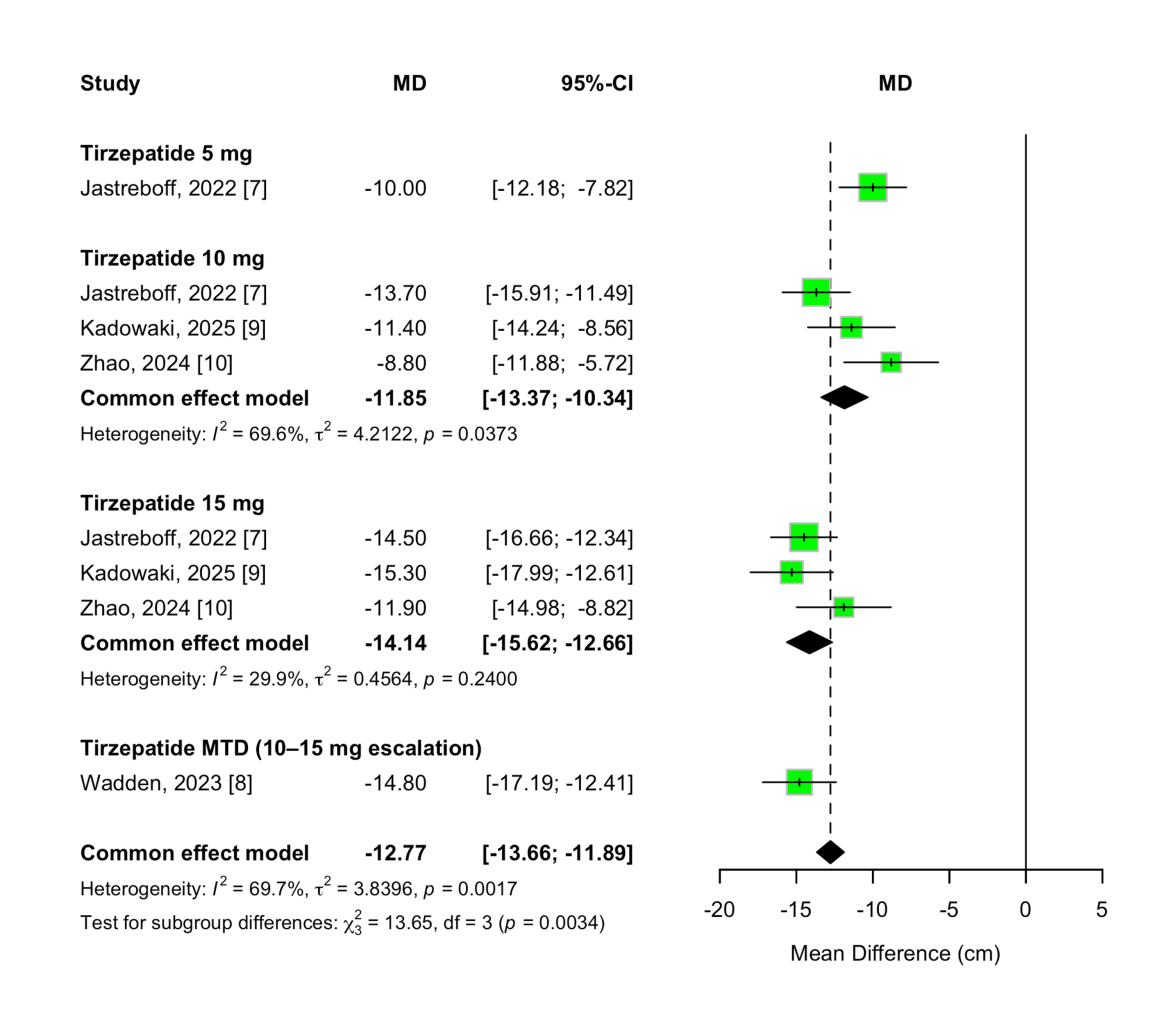
Forest plot for waist circumference change

A dose-dependent effect was observed, with greater reductions in waist circumference at higher tirzepatide doses. Treatment with 5 mg resulted in a mean reduction of -10.00 cm (95% CI -12.18 to -7.82; p < 0.00001), while 10 mg and 15 mg doses produced reductions of -11.85 cm (95% CI, -13.37 to -10.34) and -14.14 cm (95% CI -15.62 to -12.66), respectively. Participants who escalated to their MTD (10 or 15 mg) experienced the largest reduction, with an MD of -14.80 cm (95% CI -17.19 to -12.41; p < 0.00001). A formal test for subgroup differences confirmed statistically significant variation across doses (χ² = 13.65, df = 3, p = 0.003; I² = 78.0%). These results are shown in Figure 7.

**Figure 8:**
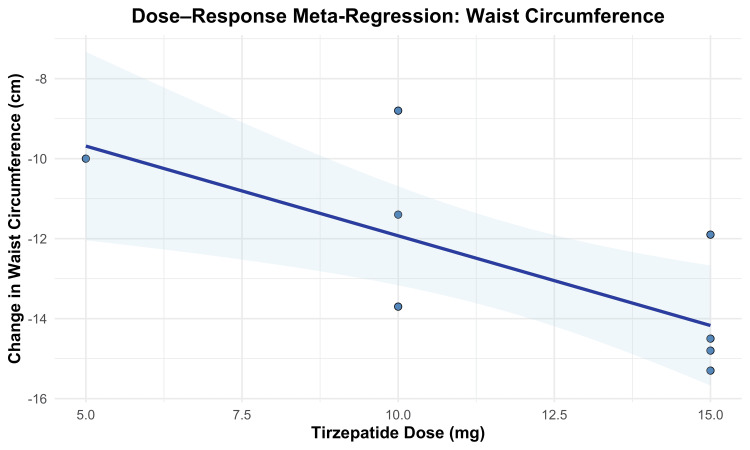
Meta-regression plot for waist circumference change

To further examine the dose-response relationship, a meta-regression analysis was conducted. Each 1 mg increase in tirzepatide dosage was associated with an additional 0.45 cm reduction in waist circumference (β = -0.4487; 95% CI -0.7565 to -0.1408; p = 0.0043). The model explained 75.85% of the between-study heterogeneity (R²), with a significant moderator effect (QM(1) = 8.1584, p = 0.0043). Residual heterogeneity was not statistically significant (QE(6) = 9.7144, p = 0.1372). The dose-response trend is illustrated in Figure 8.

HbA1c Change

Although participants did not have diabetes, changes in HbA1c were assessed as a secondary outcome to evaluate metabolic effects. Tirzepatide treatment was associated with significant reductions in HbA1c compared to placebo across all evaluated doses. The overall MD was -0.42% (95% CI -0.44 to -0.40; p < 0.00001), favoring tirzepatide. Substantial heterogeneity was observed across studies (I² = 85%, p < 0.00001).

**Figure 9:**
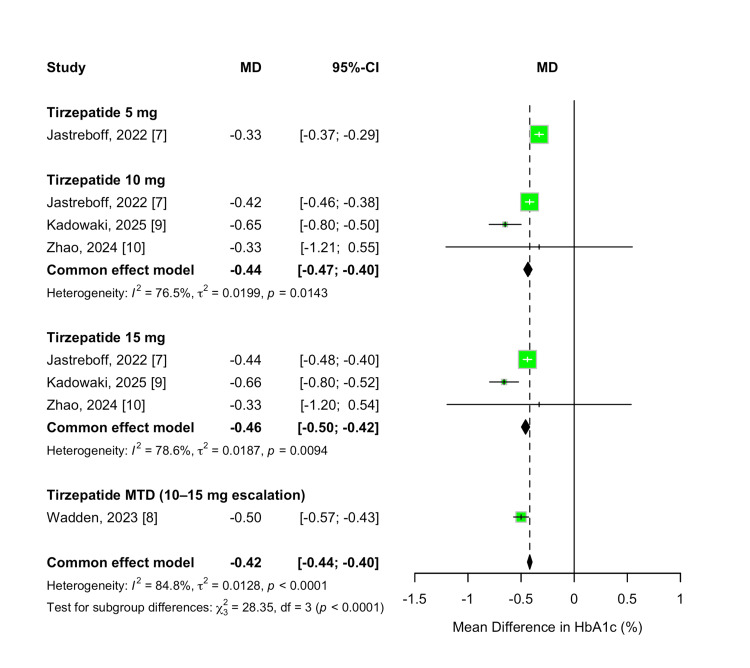
Forest plot for HbA1c change

A dose-dependent effect was evident, with greater reductions in HbA1c observed at higher tirzepatide doses. The 5 mg dose resulted in a mean reduction of -0.33% (95% CI -0.37 to -0.29; p < 0.00001), while the 10 mg and 15 mg doses produced reductions of -0.44% (95% CI -0.47 to -0.40) and -0.46% (95% CI -0.50 to -0.42), respectively. Participants who escalated to their MTD (10 or 15 mg) experienced the largest reduction, with an overall MD of -0.50% (95% CI -0.57 to -0.43; p < 0.00001). A formal test for subgroup differences confirmed statistically significant variation in HbA1c reduction across doses (χ² = 28.35, df = 3, p < 0.00001; I² = 89.4%). The results are demonstrated in Figure 9.

**Figure 10:**
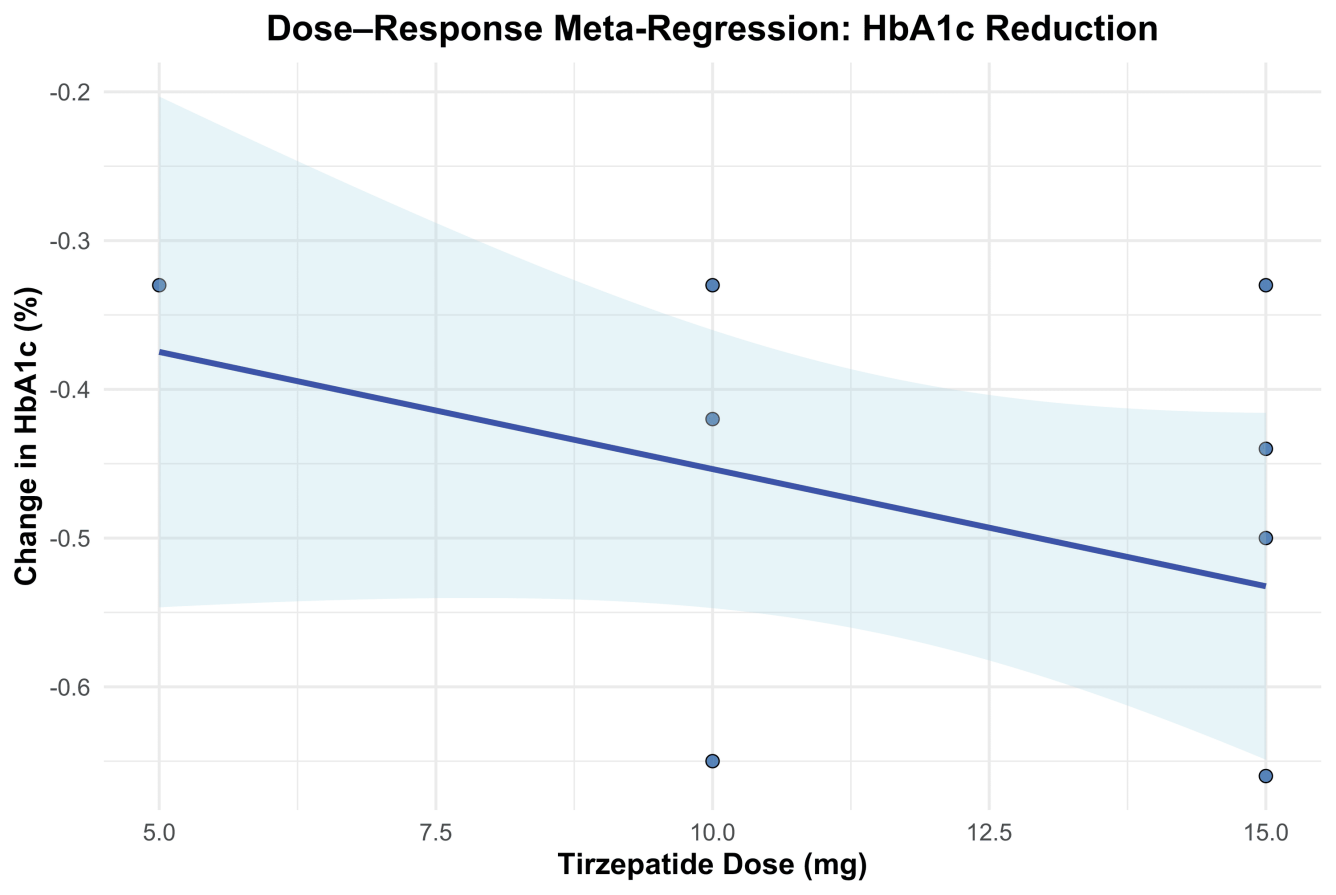
Meta-regression plot for HbA1c change

To further explore the dose-response relationship for HbA1c reduction, a meta-regression analysis was performed. Each 1 mg increase in tirzepatide dose was associated with a non-significant additional reduction of 0.016% in HbA1c (β = -0.0158; 95% CI: -0.0384 to 0.0069; p = 0.1723). The model explained 20.31% of between-study heterogeneity (R²), but the moderator effect of dose did not reach statistical significance (QM(1) = 1.86, p = 0.1723). Residual heterogeneity remained significant (QE(6) = 20.89, p = 0.0019), suggesting that other factors may contribute to the observed variability. The dose-response relationship is depicted in Figure 10.

SBP Change

The change in SBP from baseline was evaluated as a secondary outcome to determine the possible cardiovascular advantages of tirzepatide. Across all evaluated doses, tirzepatide was associated with statistically significant reductions in SBP compared to placebo. The pooled MD was -7.48 mmHg (95% CI -8.37 to -6.59; p < 0.00001), favoring tirzepatide, with substantial heterogeneity observed among studies (I² = 75%, p = 0.001).

**Figure 11:**
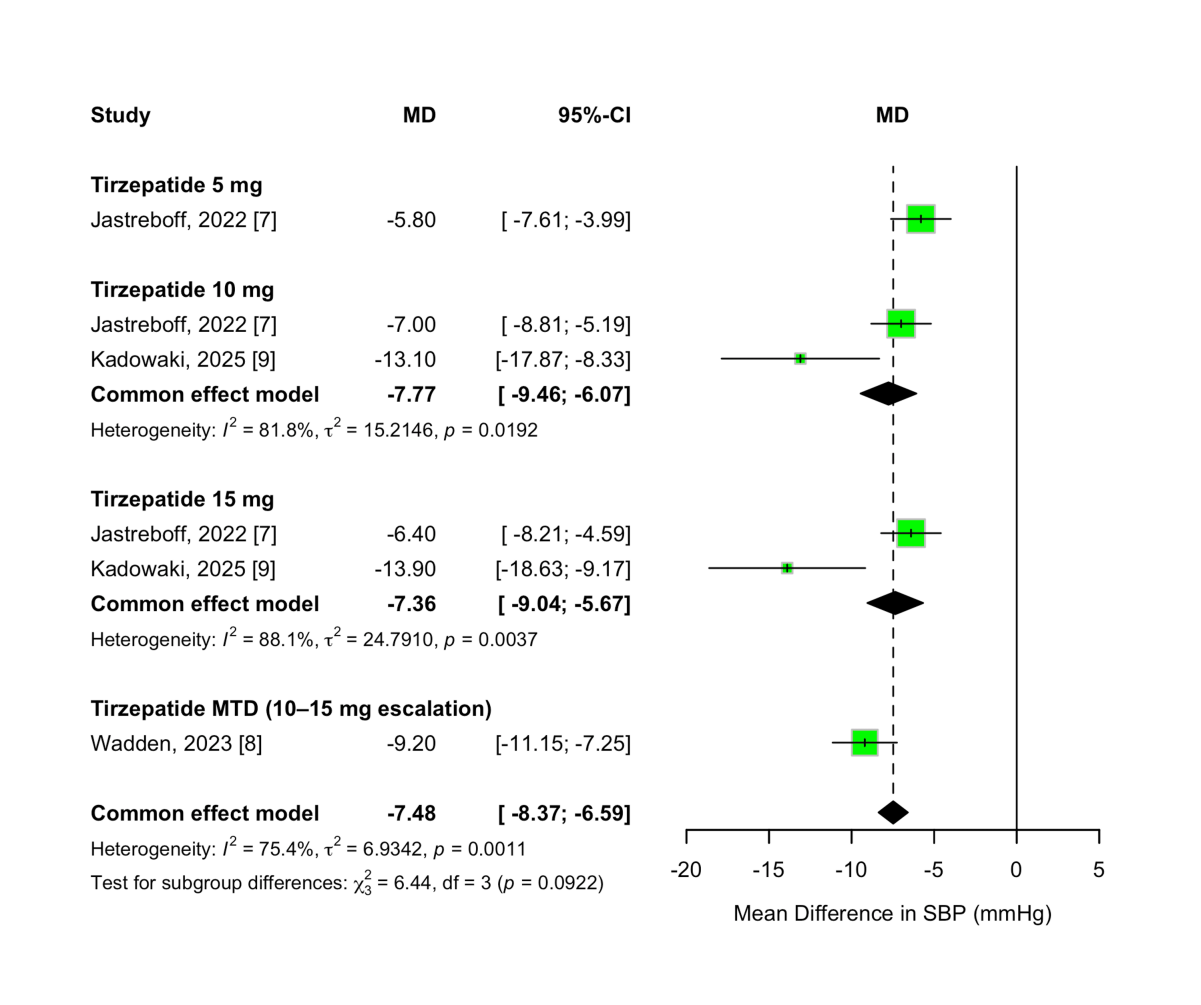
Forest plot for systolic blood pressure change

A dose-dependent trend was observed, with greater SBP reductions at higher tirzepatide doses. The 5 mg dose resulted in a mean reduction of -5.80 mmHg (95% CI -7.61 to -3.99; p < 0.00001), while the 10 mg and 15 mg doses produced reductions of -7.77 mmHg (95% CI -9.46 to -6.07) and -7.36 mmHg (95% CI -9.04 to -5.67), respectively. Participants who escalated to their MTD (10 or 15 mg) experienced the greatest reduction, with a pooled MD of -9.20 mmHg (95% CI: -11.15 to -7.25; p < 0.00001). While a trend based on dosage was apparent, the formal analysis for differences between subgroups did not achieve statistical significance (χ² = 6.44, df = 3, p = 0.09; I² = 53.4%). These results are shown in Figure 11.

**Figure 12:**
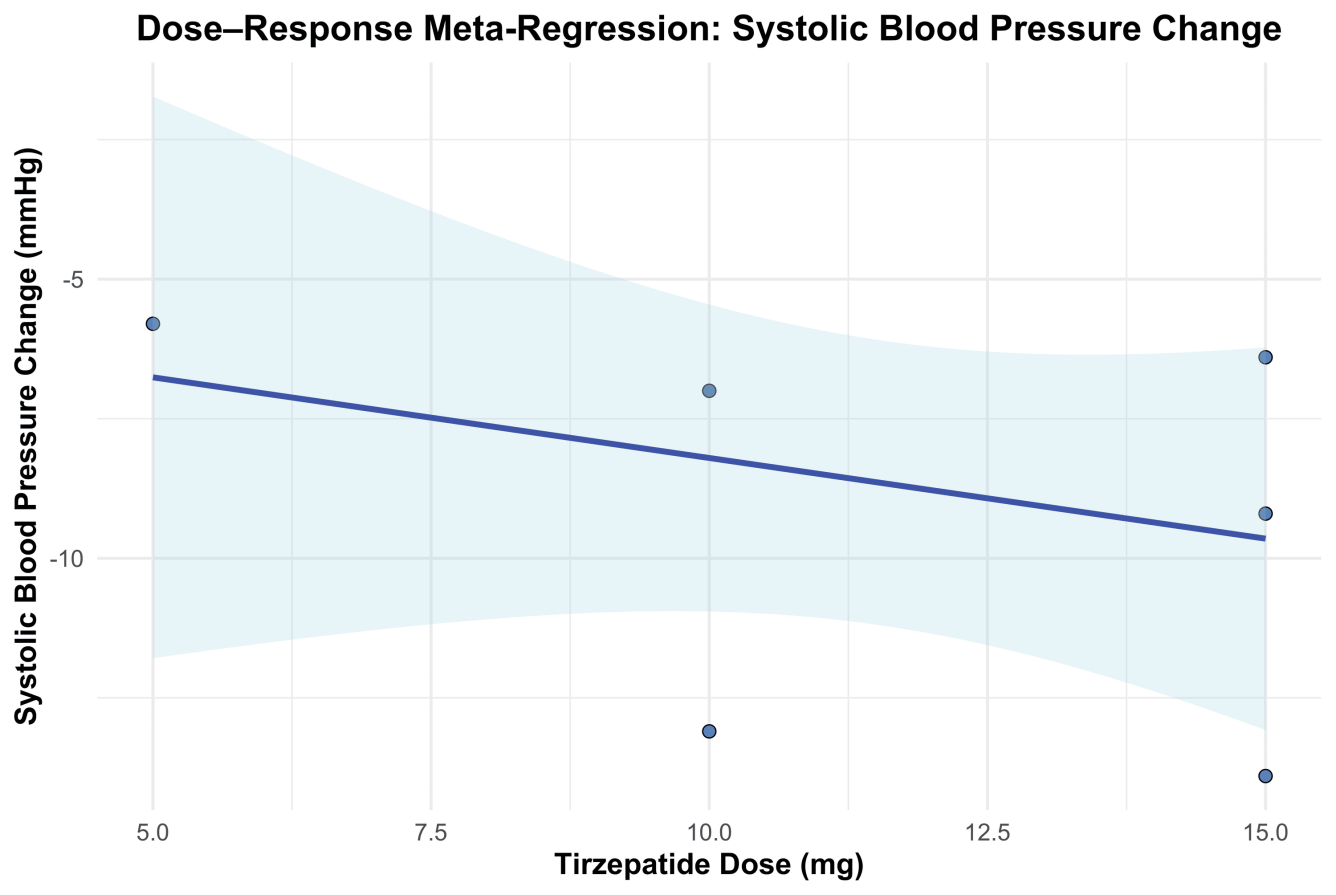
Meta-regression plot for systolic blood pressure change

To further evaluate the dose-response relationship for SBP reduction, a meta-regression analysis was conducted. Each 1 mg increase in tirzepatide dose was associated with a non-significant reduction of 0.29 mmHg in SBP (β = -0.2889; 95% CI, -0.9511 to 0.3733; p = 0.3925). The model did not explain any of the between-study heterogeneity (R² = 0%), and the moderator effect of dose was not statistically significant (QM(1) = 0.73, p = 0.3925). Residual heterogeneity remained statistically significant (QE(4) = 16.47, p = 0.0025), indicating that a substantial portion of variability across studies remained unexplained. The dose-response relationship is shown in Figure 12.

Health-Related Quality of Life (SF-36v2) Change

Health-related quality of life was assessed as a secondary efficacy outcome using the SF-36v2 questionnaire. Tirzepatide treatment resulted in significant improvements in SF-36v2 scores compared to placebo across all evaluated doses. The overall MD was 2.30 points (95% CI 1.84 to 2.75; p < 0.00001), favoring tirzepatide, with low heterogeneity across studies (I² = 39%, p = 0.12).

**Figure 13:**
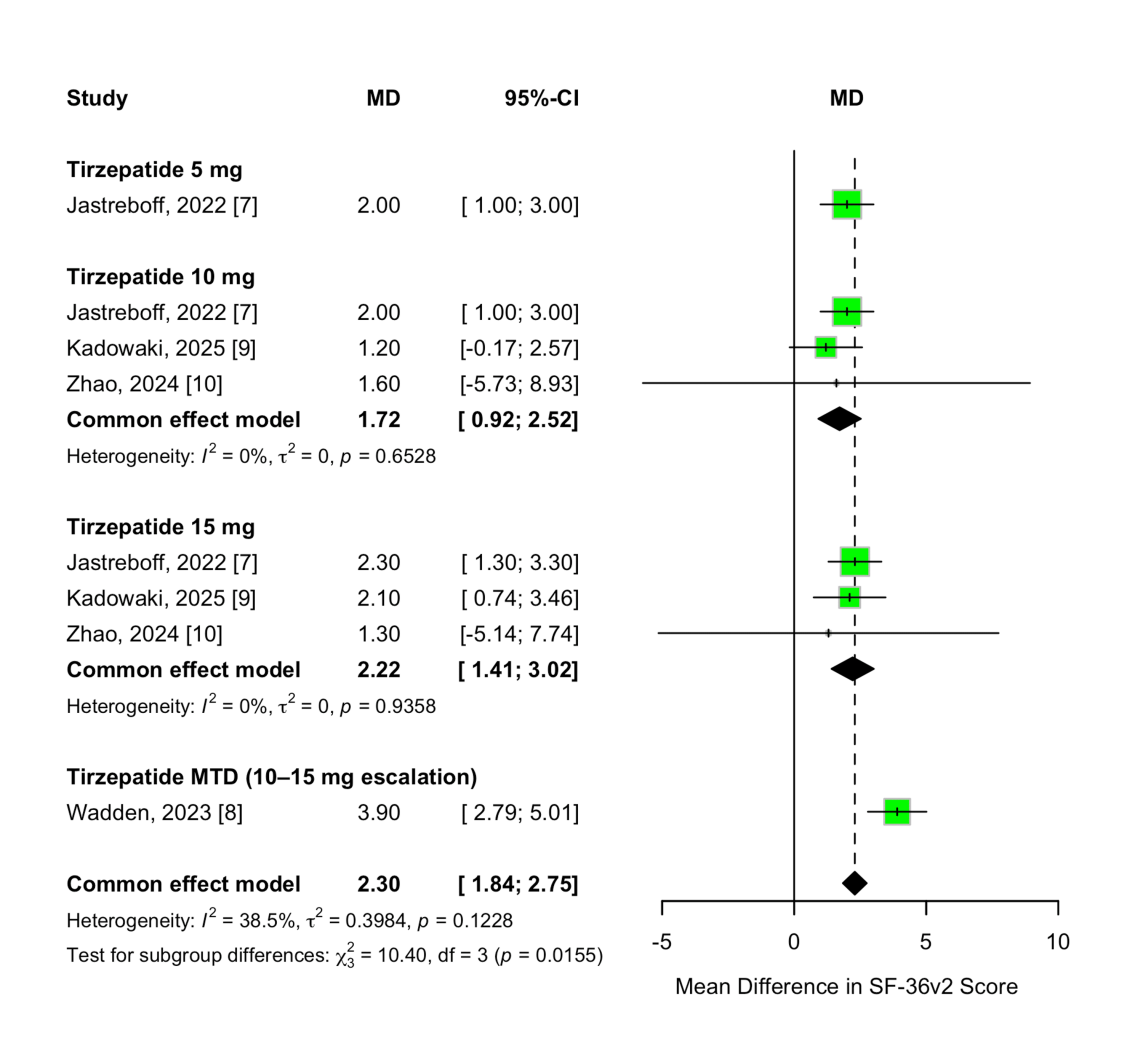
Forest plot for SF-36v2 score change SF-36v2: Short-Form Health Survey version 2.

A dose-dependent trend was observed, with greater improvements in quality-of-life scores at higher tirzepatide doses. Treatment with 5 mg resulted in a mean improvement of 2.00 points (95% CI 1.00-3.00; p < 0.0001), while the 10 mg and 15 mg doses produced improvements of 1.72 points (95% CI 0.92-2.52) and 2.22 points (95% CI 1.41-3.02), respectively. Participants who escalated to their MTD (10 or 15 mg) experienced the greatest benefit, with an MD of 3.90 points (95% CI 2.79-5.01; p < 0.00001). A formal test for subgroup differences confirmed statistically significant variation in outcomes across dose groups (χ² = 10.40, df = 3, p = 0.02; I² = 71.1%). These results are illustrated in Figure 13.

No dose-response meta-regression analysis was conducted for SF-36v2 scores due to the limited number of available study arms and the relatively low between-study variability.

Weight-Related Quality of Life (IWQOL-Lite-CT) Change

Weight-related quality of life was evaluated as a secondary efficacy outcome using the IWQOL-Lite-CT instrument. Tirzepatide treatment led to significant improvements in IWQOL-Lite-CT scores compared to placebo across all evaluated doses. The overall MD was 12.37 points (95% CI 9.75-14.99; p < 0.00001), favoring tirzepatide, with no heterogeneity observed across studies (I² = 0%, p = 0.95).

**Figure 14:**
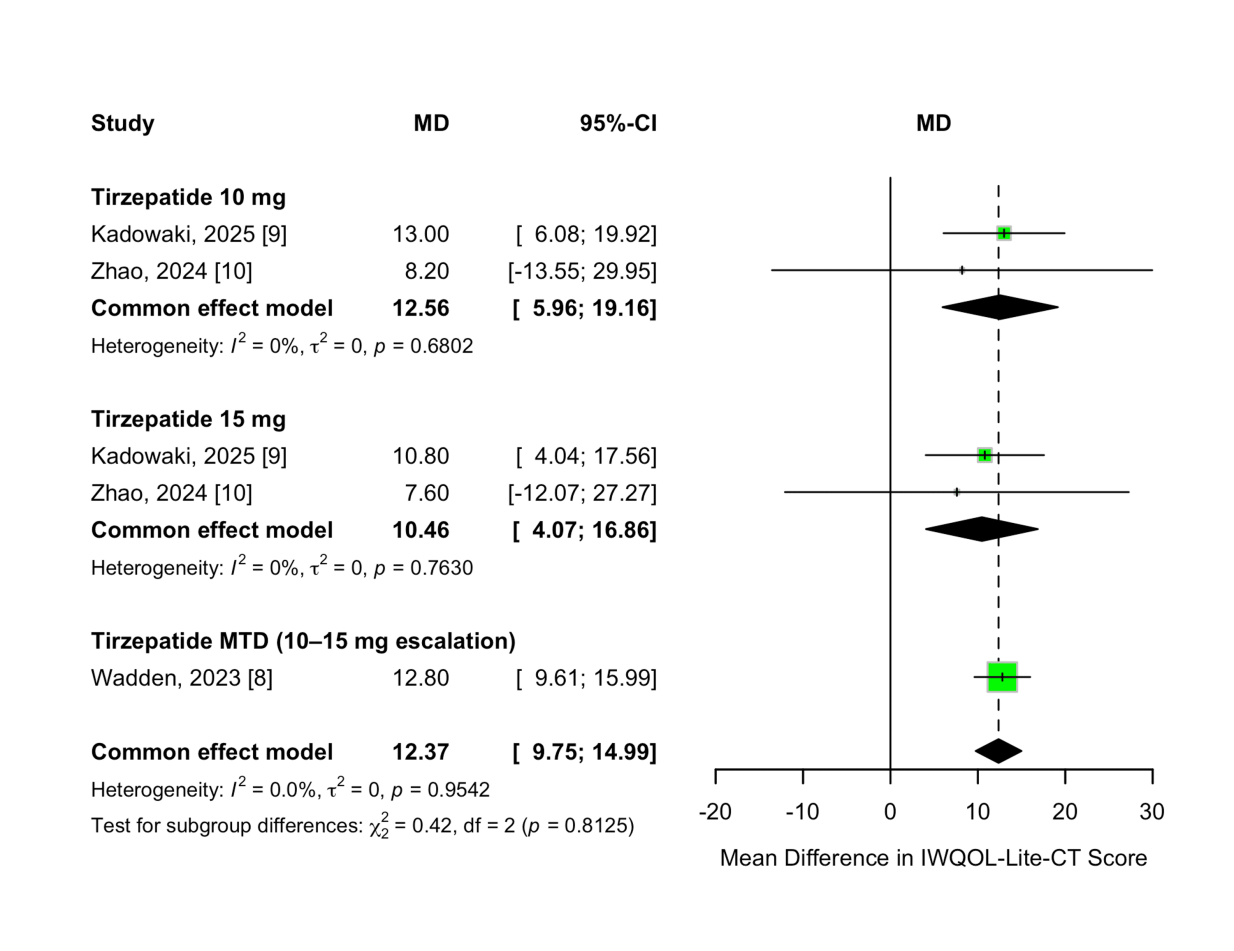
Forest plot for IWQOL-Lite-CT score change IWQOL-Lite-CT: Impact of Weight on Quality of Life-Lite Clinical Trials version.

A dose-dependent trend was observed, with higher tirzepatide doses associated with greater improvements in quality-of-life scores. The 10 mg dose resulted in a mean improvement of 12.56 points (95% CI 5.96-19.16; p = 0.0002), while the 15 mg dose produced an improvement of 10.46 points (95% CI 4.07-16.86; p = 0.001). Participants who escalated to their MTD (10 or 15 mg) experienced the greatest benefit, with a pooled MD of 12.80 points (95% CI 9.61-15.99; p < 0.00001). A formal test for subgroup differences found no statistically significant variation across dose groups (χ² = 0.42, df = 2, p = 0.81; I² = 0%). These results are demonstrated in Figure 14.

No dose-response meta-regression analysis was conducted for IWQOL-Lite-CT scores due to the limited number of available study arms and the relatively low between-study variability.

Achievement of ≥5%, ≥10%, ≥15%, ≥20%, and ≥25% Weight Loss

The proportion of participants achieving clinically meaningful categorical weight-loss thresholds (≥5%, ≥10%, ≥15%, ≥20%, and ≥25% reduction from baseline body weight) was assessed as a binary efficacy outcome. Tirzepatide significantly increased the likelihood of achieving each weight-loss target compared to placebo across all evaluated doses. 

For the ≥5% weight-loss threshold, tirzepatide was associated with a pooled OR of 19.62 (95% CI 16.37-23.52; p < 0.00001). For the ≥10% threshold, the OR was 18.60 (95% CI 15.38-22.50; p < 0.00001). Achievement of ≥15% weight loss yielded an OR of 23.25 (95% CI 18.06-29.94; p < 0.00001), while the ≥20% threshold produced an OR of 32.14 (95% CI 21.59-47.84; p < 0.00001). For the most stringent threshold, ≥25% weight loss, the OR was 25.57 (95% CI 14.96-43.70; p < 0.00001). 

**Figure 15:**
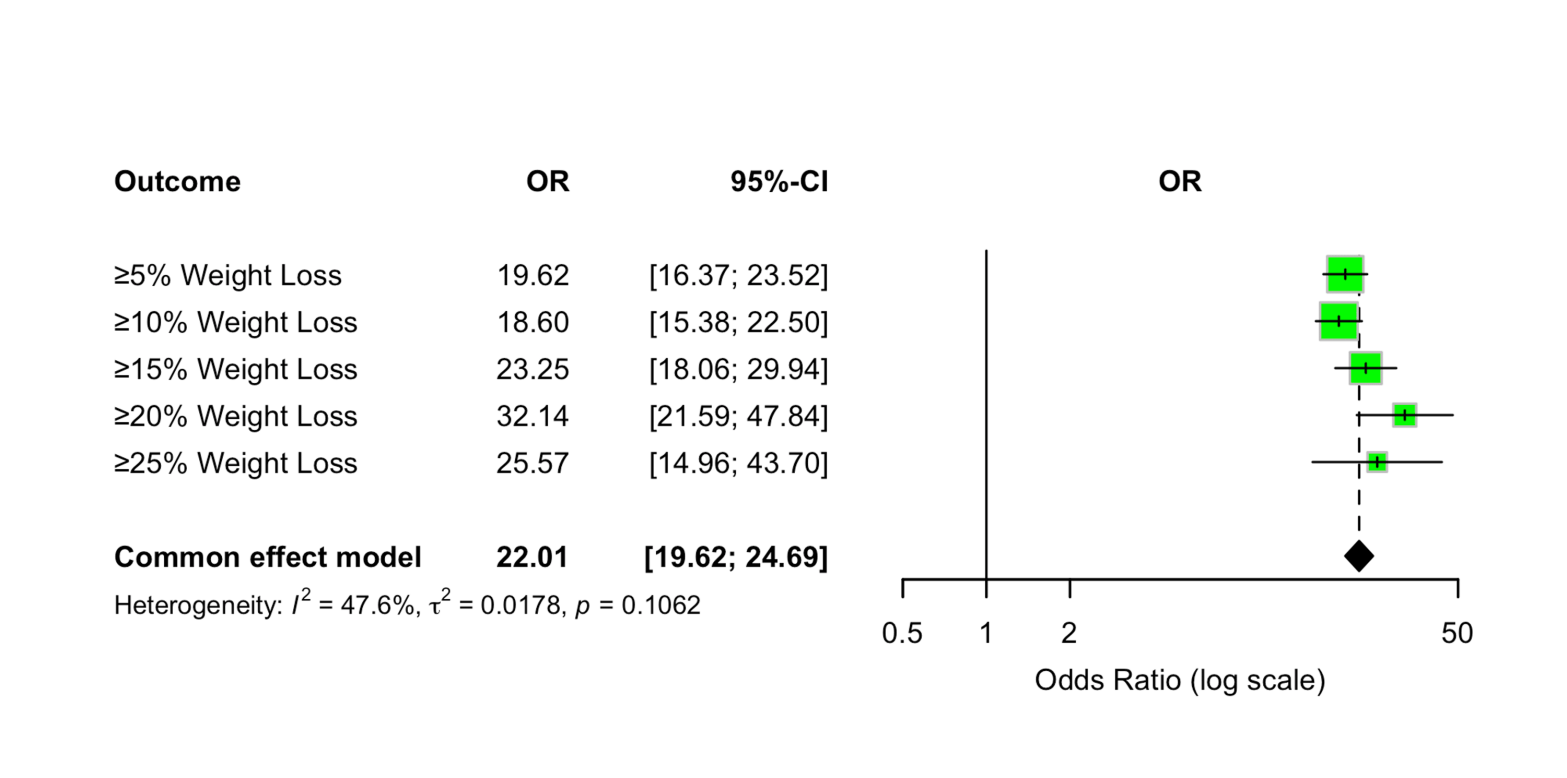
Forest plot for achievement of ≥5%, ≥10%, ≥15%, ≥20%, and ≥25% weight loss

Overall heterogeneity across these categorical outcomes was moderate (I² = 48%). The pooled OR across all thresholds strongly favored tirzepatide over placebo, with a combined OR of 22.01 (95% CI 19.62-24.69). Due to the categorical nature of the outcomes, no dose-response meta-regression analysis was performed. These results are presented in Figure 15.

Overall Safety Outcomes

**Figure 16:**
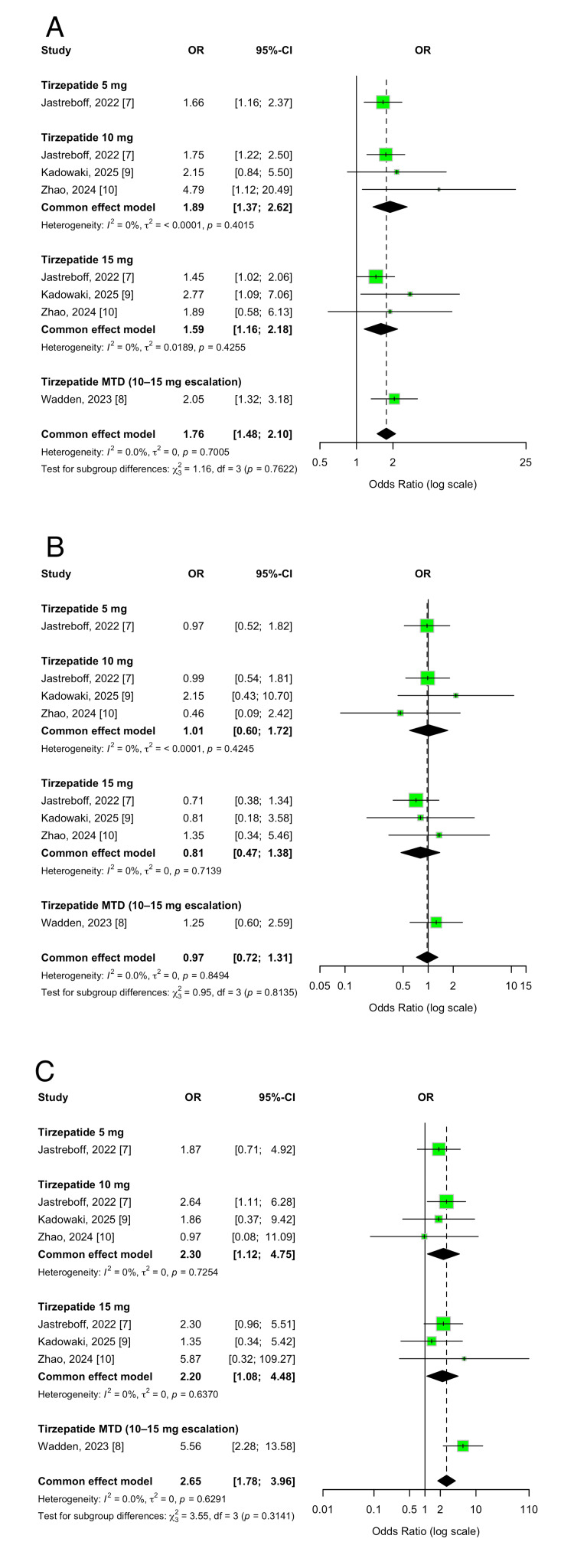
Forest plots for (A) any adverse events, (B) serious adverse events, and (C) discontinuations due to adverse events

Tirzepatide treatment was associated with a significantly higher incidence of any adverse events when compared to placebo (OR 1.76; 95% CI 1.48-2.10; p < 0.00001). Most adverse events were mild to moderate in severity and consistent with the known tolerability profile of GLP-1 receptor agonists. The incidence of serious adverse events was comparable between the tirzepatide and placebo groups (OR 0.97; 95% CI 0.72-1.31; p = 0.85), with low heterogeneity (I² = 0%), suggesting no increased risk of serious toxicity over the 52-72 week treatment period. However, discontinuations due to adverse events were more common among participants receiving tirzepatide than among those receiving placebo (OR 2.65; 95% CI 1.78-3.96; p < 0.00001), likely reflecting differences in tolerability, particularly gastrointestinal-related symptoms. These findings are illustrated in Figure 16.

Nausea

**Figure 17:**
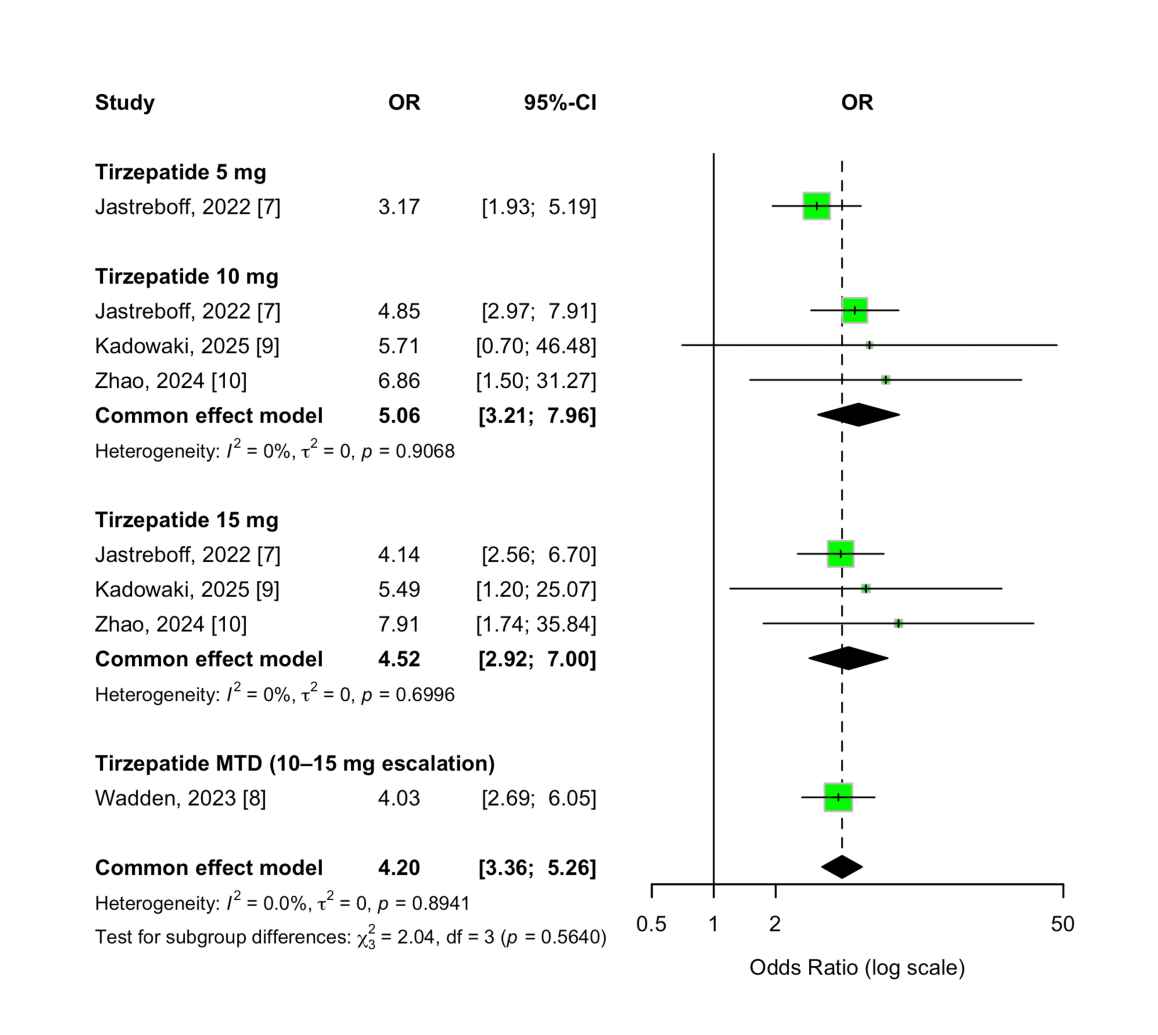
Forest plot for nausea events

The incidence of nausea was significantly higher among participants treated with tirzepatide compared to those receiving a placebo. The overall OR was 4.20 (95% CI 3.36-5.26; p < 0.00001), indicating a strong association with this gastrointestinal side effect. Across all evaluated doses, tirzepatide consistently increased the risk of nausea, with no evidence of between-study heterogeneity (I² = 0%). Subgroup analysis showed overlapping effect estimates for the 5 mg, 10 mg, 15 mg, and MTD groups, with no significant difference observed between dose levels (p-value for subgroup differences = 0.56). These results are shown in Figure 17.

Vomiting

**Figure 18:**
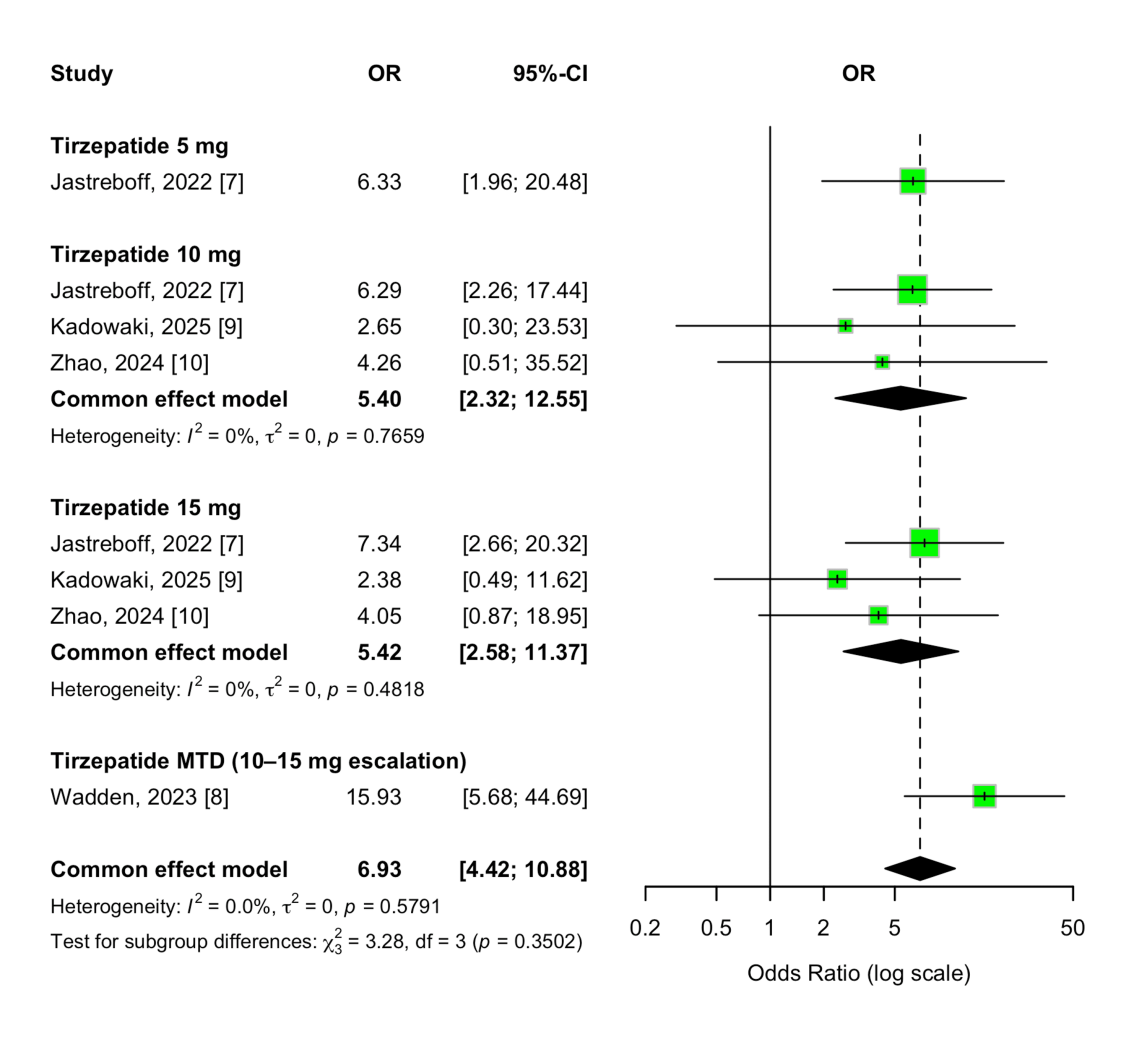
Forest plot for vomiting events

The incidence of vomiting was also significantly elevated in participants treated with tirzepatide compared to those receiving a placebo. The pooled OR was 6.93 (95% CI, 4.42-10.88; p < 0.00001), reflecting a strong association with gastrointestinal tolerability. This effect was consistent across all evaluated doses (5 mg, 10 mg, 15 mg, and MTD), with overlapping CIs and no significant between-group differences (p-value for subgroup differences = 0.35). Heterogeneity across studies was minimal (I² = 0%), indicating a robust and reproducible adverse event profile. These findings are illustrated in Figure 18.

Diarrhea

**Figure 19:**
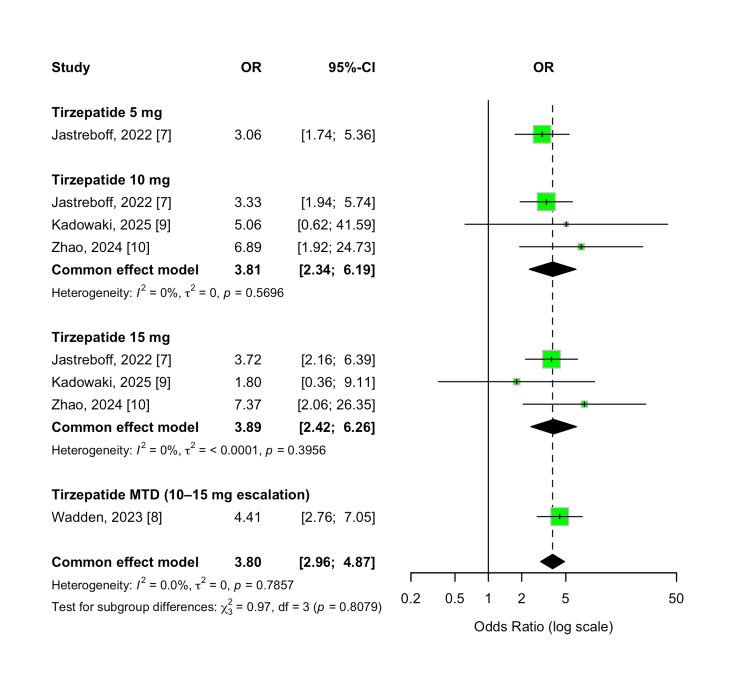
Forest plot for diarrhea events

Tirzepatide was also associated with a significantly increased risk of diarrhea compared to placebo, with an overall OR of 3.80 (95% CI 2.96-4.87; p < 0.00001). This effect was observed consistently across all tirzepatide dose groups (5 mg, 10 mg, 15 mg, and MTD) with no significant variation between doses (p = 0.81). Between-study heterogeneity was negligible (I² = 0%), supporting a robust and reproducible pattern of gastrointestinal disturbance. These findings are presented in Figure 19.

Sensitivity Analyses

Leave-one-out sensitivity analyses were performed to evaluate how individual studies impacted the overall estimates for all primary and secondary outcomes. The results were stable throughout the analyses. For the primary efficacy outcome, percent change in body weight, removal of any one study did not meaningfully alter the pooled estimate, which ranged from -15.71% to -17.11%, with consistently high heterogeneity (I² = 80%-88%). Similar stability was observed for secondary outcomes, including BMI reduction (-5.27 to -6.59 kg/m²; I² = 84.5%-95.9%) and waist circumference (-12.27 to -13.11 cm; I² = 62%-75%). Consistent results were also noted for SBP (-7.67 to -9.28 mmHg; I² = 73%-88%) and HbA1c change (-0.45% to -0.51%; I² = 84.7%-93.6%), as well as for both quality-of-life measures: SF-36v2 (1.98-2.43 points; I² = 0%-55%) and IWQOL-Lite-CT (11.48-12.65 points; I² = 0%).

Binary responder outcomes for weight-loss thresholds (≥5% to ≥25%) also demonstrated consistent results. Pooled ORs varied minimally with study exclusion. Heterogeneity was moderate to high for the lower thresholds (I² = 66%-85%) but decreased at higher thresholds, reaching 0% for the ≥25% weight-loss category. Safety and tolerability outcomes showed similarly strong consistency. No single study significantly influenced the pooled estimates for any adverse events (OR 0.54-0.63; I² = 0%) or treatment discontinuations due to adverse events (OR -0.09 to +0.04; I² = 0%). Gastrointestinal side effects, including nausea (OR 1.39-1.49), vomiting (OR 1.66-1.97), and diarrhea (OR 1.28-1.39), remained stable across iterations, with minimal or no heterogeneity. Overall, these findings confirm that the study’s conclusions are robust and not driven by any single trial.

Discussion

Summary of Main Findings

This systematic review and meta-analysis of RCTs show that tirzepatide induces substantial and clinically meaningful weight loss in obese adults who do not have diabetes. Across all evaluated doses, tirzepatide significantly reduced the percent body weight, BMI, and waist circumference, with a clear dose-response relationship supported by subgroup and meta-regression analyses. A large proportion of participants achieved key weight-loss milestones (≥5% to ≥25%), and treatment was also associated with improvements in both general (SF-36v2) and obesity-specific (IWQOL-Lite-CT) quality of life. Although gastrointestinal side effects, including nausea, vomiting, and diarrhea, were more common among those receiving tirzepatide, the incidence of serious adverse events was comparable to placebo, and overall tolerability remained acceptable. Sensitivity analyses confirmed that these findings were robust across all outcomes and not driven by any single study.

Comparison With Prior Literature

The findings of this meta-analysis align with and build upon results from individual SURMOUNT trials, which have demonstrated that tirzepatide induces significant weight loss in both diabetic and non-diabetic populations [7-10]. Compared to GLP-1 receptor agonists such as semaglutide and liraglutide, tirzepatide appears to offer greater weight reduction, likely attributable to its dual agonism of both GIP and GLP-1 receptors [5,15]. Prior meta-analyses of GLP-1 monotherapy in non-diabetic individuals have typically reported more modest weight loss in the range of 6% to 10%. In contrast, this analysis observed pooled reductions exceeding 16%, with notably higher odds of achieving ≥15% or ≥20% weight loss than those reported for current monotherapies [16]. Moreover, the dose-dependent efficacy observed in this meta-analysis reinforces findings from the phase 3 SURMOUNT program and underscores the therapeutic importance of dose escalation when clinically appropriate and well tolerated. While gastrointestinal adverse events remain the most common limiting factor to tolerability, the lack of an observed increase in serious adverse events or treatment-related mortality is consistent with the safety profiles reported in previous studies of incretin-based therapies [17].

Interpretation and Clinical Significance

This meta-analysis highlights the significant clinical value of tirzepatide in managing obesity among non-diabetic adults. The degree of weight loss, mainly the high odds of achieving ≥15%, ≥20%, or even ≥25% reduction from baseline, approaches outcomes typically seen with bariatric surgery. For context, the STAMPEDE trial reported mean weight reductions of approximately 23% following gastric bypass and 19% after sleeve gastrectomy at five years of follow-up [3]. Such efficacy is especially remarkable given that this analysis focused on non-diabetic individuals, a population often underrepresented in trials of metabolic therapies. The observed dose-response relationship underscores the clinical importance of individualized titration to each patient’s MTD, optimizing both efficacy and long-term adherence. These results position tirzepatide not only as a strong second-line option following lifestyle modification but also as a potential first-line pharmacologic treatment for patients with severe obesity or obesity-related comorbidities.

Beyond weight metrics, the improvements in both general (SF-36v2) and obesity-specific (IWQOL-Lite-CT) quality-of-life outcomes suggest that tirzepatide may also enhance functional status and psychosocial well-being. While gastrointestinal adverse events were common, they were generally mild to moderate in severity and matched the known profile of incretin-based therapies. The low incidence of serious adverse events and strong treatment retention support its overall tolerability in this population. These findings support the expanded use of tirzepatide in the treatment of non-diabetic obesity and suggest that it may serve as a viable non-surgical alternative for individuals who would otherwise be considered for bariatric intervention.

Mechanistic Insight

The superior efficacy of tirzepatide compared to traditional GLP-1 receptor agonists is likely explained by its dual mechanism of action. Tirzepatide targets both the GIP and GLP-1 receptors, thereby mimicking the actions of two key endogenous incretin hormones involved in appetite regulation, insulin secretion, and metabolic homeostasis. GLP-1 receptor activation enhances satiety, slows gastric emptying, and stimulates insulin release in a glucose-dependent manner. GIP receptor activation, meanwhile, is believed to amplify insulinotropic effects and contribute to improved lipid metabolism and fat utilization. Preclinical studies suggest that simultaneous activation of both receptors in a single molecule may produce additive or even synergistic effects on weight reduction and glycemic regulation, surpassing the efficacy of GLP-1 monotherapy [6]. This dual-incretin mechanism probably explains the strong and dose-dependent weight-loss results seen in this meta-analysis, offering a mechanistic justification for the improved clinical efficacy of tirzepatide.

Strengths

This meta-analysis offers several important strengths. First, it represents the most current and comprehensive synthesis of tirzepatide’s efficacy and safety in obese adults who do not have diabetes, a population increasingly considered in clinical decision-making yet historically underrepresented in obesity pharmacotherapy trials. Second, the analysis included only RCTs with a minimum duration of 52 weeks, providing high-quality evidence relevant to long-term treatment outcomes. Third, the study evaluated a wide range of outcomes, including both continuous and categorical measures of weight loss, covering clinically meaningful thresholds from ≥5% to ≥25%. It also incorporated quality-of-life assessments and cardiometabolic parameters, offering a holistic view of treatment impact. The dose-response meta-regression further strengthened the findings by confirming a consistent relationship between tirzepatide dose and the magnitude of effect. Finally, sensitivity analyses across all primary and secondary outcomes demonstrated the robustness of the results, confirming that no single study unduly influenced the conclusions.

Limitations

This meta-analysis has a few limitations to consider. First, although all included studies were RCTs, they were sponsored by the same pharmaceutical manufacturer, which may raise sponsorship bias despite rigorous methodologies and consistent reporting practices. Second, the analysis was limited to trials with follow-up durations of at least 52 weeks. While this enhances the relevance to long-term treatment outcomes, it excludes shorter-duration studies that may still provide valuable insights into early safety signals and dose-titration effects. Third, while all participants met inclusion criteria for obesity and absence of diabetes, variability in baseline characteristics, such as age, sex distribution, comorbidities, and geographic region, may have contributed to heterogeneity between studies. Fourth, adverse gastrointestinal events were mainly self-reported, which may introduce inconsistencies or underreporting, limiting the precision of tolerability assessments. Finally, the relatively small number of eligible trials limited the ability to formally assess publication bias and restricted subgroup analyses beyond dose stratification. *Future Directions*

Several areas require further investigation. Extended follow-up beyond 72 weeks is needed to evaluate the long-term durability of tirzepatide-induced weight loss and its effects on clinically meaningful outcomes such as cardiovascular events, mortality, and obesity-related complications. Head-to-head trials comparing tirzepatide with other anti-obesity medications, including semaglutide and emerging multi-agonist therapies, will be essential to determine its relative efficacy and safety.

Additionally, future studies should assess real-world effectiveness across diverse patient populations, including individuals with multiple comorbidities, varying socioeconomic status, and different racial and ethnic backgrounds. Cost-effectiveness evaluations and implementation research will be crucial in shaping policy choices and facilitating the incorporation of tirzepatide into conventional obesity treatment.

## Conclusions

In obese adults without diabetes, tirzepatide provides substantial, dose-dependent weight loss, accompanied by improvements in quality of life and an acceptable safety profile. These findings position tirzepatide as a leading pharmacologic option for the treatment of obesity, particularly for individuals who are not candidates for bariatric surgery or who prefer non-invasive alternatives. As clinical use of dual-incretin therapies continues to expand, future research focused on long-term outcomes, comparative effectiveness, and equitable access will be critical to maximizing their impact on obesity management.
